# A Deep Learning Pipeline to Automate High-Resolution Arterial Segmentation With or Without Intravenous Contrast

**DOI:** 10.1097/SLA.0000000000004595

**Published:** 2020-11-23

**Authors:** Anirudh Chandrashekar, Ashok Handa, Natesh Shivakumar, Pierfrancesco Lapolla, Raman Uberoi, Vicente Grau, Regent Lee

**Affiliations:** * Nuffield Department of Surgical Sciences, University of Oxford, Oxford, United Kingdom; † Department of Engineering Science, University of Oxford, Oxford, United Kingdom.

**Keywords:** aorta, aortic aneurysm, attention-gating, CT angiogram, deep learning, segmentation, U-Net

## Abstract

Existing methods to reconstruct vascular structures from a computerized tomography (CT) angiogram rely on contrast injection to enhance the radio-density within the vessel lumen. However, pathological changes in the vasculature may be present that prevent accurate reconstruction. In aortic aneurysmal disease, a thrombus adherent to the aortic wall within the expanding aneurysmal sac is present in >90% of cases. These deformations prevent the automatic extraction of vital clinical information by existing image reconstruction methods.

**Aim:**

In this study, a deep learning architecture consisting of a modified U-Net with attention-gating was implemented to establish a high-throughput and automated segmentation pipeline of pathological blood vessels in CT images acquired with or without the use of a contrast agent.

**Methods and Results:**

Seventy-Five patients with paired noncontrast and contrast-enhanced CT images were randomly selected from an ongoing study (Ethics Ref 13/SC/0250), manually annotated and used for model training and evaluation. Data augmentation was implemented to diversify the training data set in a ratio of 10:1. The performance of our Attention-based U-Net in extracting both the inner (blood flow) lumen and the wall structure of the aortic aneurysm from CT angiograms was compared against a generic 3-D U-Net and displayed superior results. Implementation of this network within the aortic segmentation pipeline for both contrast and noncontrast CT images has allowed for accurate and efficient extraction of the morphological and pathological features of the entire aortic volume.

**Conclusions:**

This extraction method can be used to standardize aneurysmal disease management and sets the foundation for complex geometric and morphological analysis. Furthermore, this pipeline can be extended to other vascular pathologies.

Acomputerized tomography (CT) scan uses multiple X-ray measurements to provide a noninvasive visualization of internal structures. Since the invention of the first commercially-available CT scanner in 1972,^1^ the use of CT for the diagnosis and disease management is extensively embedded in modern medicine. Visualization of vasculature on a routine CT is challenging as vessels have similar radio-densities (measured in Hounsfield Unit, HU) to adjacent soft tissues. Injection of intravenous contrast enhances the radio-density within the vessel, enables its visualization and permits rapid segmentation. The produced CT angiogram (CTA) is routinely utilized for diagnosis. On the other hand, vascular segmentation from noncontrast CT images is a time-intensive and challenging task. Such methods are not readily available to clinicians.

Furthermore, pathological changes, present in the lumen, vessel wall or a combination of both, impede automatic segmentation. In the example of abdominal aortic aneurysms (AAA, abnormal ballooning of the aorta) (Fig. S1A, http://links.lww.com/SLA/C720, red arrow), a thrombus is adherent to the aneurysmal aortic wall (Fig. S1B, http://links.lww.com/SLA/C720, red arrow points toward the AAA) in >90% of cases.^2^ Existing methods to segment these CTAs are unable to consistently extract the thrombus and the complex thrombus-lumen interface with accuracy. As such, no automated and standardized methods exist to assess aneurysmal diameter (Fig. S1C, http://links.lww.com/SLA/C720) or thrombus volume. These are vital pieces of clinical information used in the care of AAA patients.

Before the advent of deep learning (DL), vascular segmentation methods incorporated traditional tools including edge detection and/or mathematical models. These methods are complex, difficult to execute and are poorly generalizable. In the early 2000s, image-based DL methods became more approachable, given significant improvements in hardware. Convolutional neural networks, which are the foundation of DL architectures, consist of multiple layers that transform the input using various predefined methods (convolution, nonlinear activation, pooling, etc). The derived high-level abstractions are then extracted by fully connected layers. Finally, the weights of each neural layer and by extension the model are optimized during training.^3,4^ In recent years, many groups have strived to identify improvements to this conventional approach.

One well-known architecture for biomedical image segmentation is the U-Net.^5^ This model employs skip connections between layers, which serve to integrate the spatial and contextual information, to assemble a more precise output. Furthermore, these methods, which were initially limited to 2D, have been applied to 3D images to fully utilize spatial information.^3,5^ However, due to memory limitations, many 3D U-Net methods utilize down-sampled input images. This input size may not have enough resolution to represent its diverse anatomical variety. This is especially relevant when evaluating structures with variation that can only be captured at higher resolutions.^3,6^ Additionally, most methods are not automatic and require complex user input.

In this study, a modified U-Net architecture was implemented to achieve high-throughput, automated segmentation of pathological vessels (AAA) in CT images acquired with or without the use of IV contrast. In CTA images, our method enables simultaneous segmentation of both the arterial wall and lumen to enable characterization of pathological contents. The model’s efficacy was demonstrated by segmenting the thoracic and abdominal aortic regions. Finally, clinical relevance of the trained models was extensively evaluated.

## Methods

### Curation of CT Images From a Clinical Cohort

Chest and abdominal CT images were acquired through the Oxford Abdominal Aortic Aneurysm (OxAAA) study. This study received full ethics approval from both Oxford University and Oxford University Hospitals (OUH) NHS Foundation Trust (Ethics Ref 13/SC/0250). As part of the routine preoperative assessment for AAA, a noncontrast CT of the abdomen and a CTA of both the chest and abdomen were performed. CTA images were obtained following contrast injection in helical mode with a predefined slice thickness of 1.25 mm. Noncontrast CT images included only the abdominal aorta and were obtained with a predefined slice thickness of 2.5 mm. Paired images were anonymized within the OUH PACS system before being downloaded onto the secure study drive.

### Manual Segmentation of CT Images (Defining the Ground Truth Data)

Seventy-Five patients with paired noncontrast and CTA images were selected. In the CTA, both the aortic lumen and wall structure (WS) were segmented from the aortic root to the iliac bifurcation using the ITK-Snap segmentation software.^7^ Semi-automatic segmentation of the aortic lumen was achieved using region-growing by manually delimiting the target intensities between the contrast-enhanced lumen and surrounding tissue. Segmentation of the wall was performed manually by drawing along its boundary using the previously obtained inner lumen as a base. Removing the lumen from the larger segmentation results in a mask highlighting the WS and intra-luminal thrombus (ILT), if present. In the noncontrast CT image, the aorta was manually segmented.

**Figure 1:**
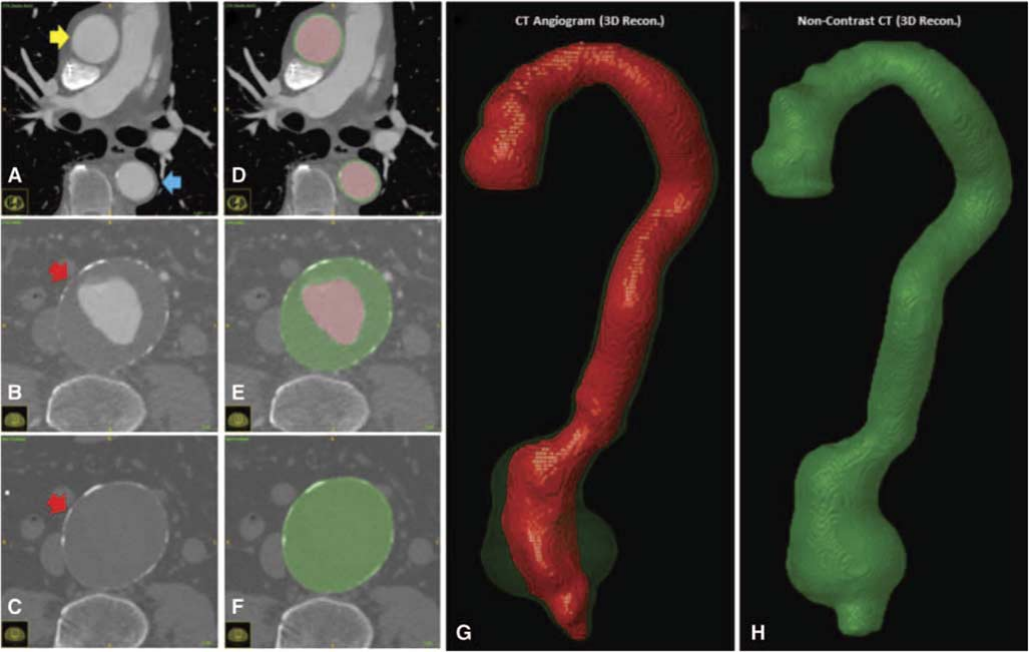
A-F. Axial Slices obtained from a CTA and noncontrast CTscan with overlaying manually segmented labels. The lumen is illustrated in black and is typically surrounded by the outer wall in grey. In the abdominal region, the grey label includes the intra-luminal thrombus, if present. G–H. 3D-reconstructed volumes representing the aortic lumen (black) and wall structure (grey), which contains the intra-luminal thrombus, are generated from the masks.

Axial CTA images depicting the ascending thoracic (*yellow arrow*), descending thoracic (*blue arrow*), and abdominal (*red arrow*) aortic regions are shown inFigure 1A-B. The latteris aneurysmal and contains crescentic layers of thrombus. Figure 1C displays the cross-section of the abdominal aorta (*red arrow*) in the noncontrast CT scan. Figure 1D–F show the CT images with the overlying manual segmentations. 3D volumes derived from the manual 2D segmentations are depicted in Figure 1G-H.

### Assessment of Intra- and Inter- Observer Variation of Manual Segmentation

Ten patients were selected for intra- and inter-observer variability evaluation. This evaluates the validity of the manual segmentations. For the intra-observer assessment, manual segmentation was performed for the second time by AC after a gap of 2 weeks. For the inter-observer assessment, a trained clinician (NS) performed the segmentations independent of the primary observer. The intraclass correlation coefficient (ICC) was calculated for the intra-/inter-observer analysis to assess the consistency of inner lumen and WS/ILT segmentations.

### Data Augmentation

To diversify the training set, CT images and their corresponding segmentations were augmented using divergence transformations. These augmentations employ non-linear warping techniques to manipulate the image in predefined locations. Each image was augmented 10:1 to obtain a total of 825 postaugmented scans. Figure S2, http://links.lww.com/SLA/C720 illustrates an axial slice augmented 10 times. During model training, images were further augmented in 3D using random rotation (0°–15°), translation and scaling (0.7–1.3).

### U-Net Architecture

**Figure 2:**
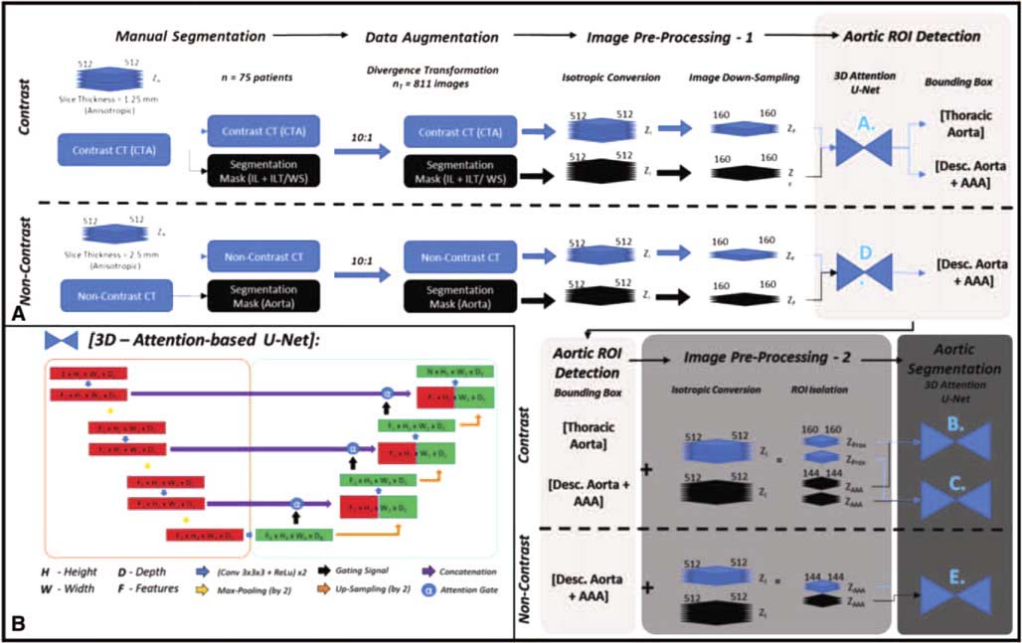
A. Automatic aortic segmentation pipeline for the simultaneous detection of the aortic lumen, and intra-luminal thrombus/wall structure. Training required manual segmentation, 2D/3D-data augmentation and preprocessing of both CTA and noncontrast CT images. Aortic ROI detection is coordinated by U-Net A for CTA images and U-Net D for noncontrast CT images. This is followed by aortic segmentation and is coordinated by U-Net B+C for CTA images and U-Net E for non-contrast CT images. B. The base architecture for this pipeline is a 3D Attention-based U-Net.

In this study, a variation of the U-Net was used for the aortic segmentation pipeline (Fig. 2A).^5,6^ Its general architecture consists of 2 components: the contraction and expansion path (Fig. 2B). The contraction path (red) extracts information to capture the context of the input at the expense of losing spatial information. This is followed by an expansion path (green), where the size of the image increases to produce a predictive binary mask. The lost image detail is restored using skip connections and is merged via concatenation. This integrates the spatial and contextual information to assemble a more precise prediction of the aortic structure.

### Attention Gating to Strengthen U-Net Performance

An attention-gated 3D U-Net was evaluated for the segmentation of the aneurysmal aorta. Attention gates utilize information extracted from the coarse scale to filter out irrelevant data exchanged via the skip connections before the concatenation step. The output of each attention gate is the element-wise multiplication of input feature-maps and a learned attention coefficient [0–1]. Given the goal to simultaneously predict the location of the aortic lumen and WS, multidimensional attention coefficients were used. These coefficients were determined using additive addition,^8^ which is more accurate than multiplicative addition.^9^ The integration of attention gates for the purpose of pancreatic segmentation has produced superior results when compared to that of prior models.^6^ A similar attention mechanism was implemented in this study for aortic segmentation. The performance of this modified U-Net architecture against that of a generic 3D U-Net for segmentation of the aneurysm is included in the supplement. Figure 2B illustrates the 3D U-Net architecture with attention gates utilized in this study.

### Loss Function to Evaluate Model Performance

The DICE score was used to quantify model performance at each step. This metric evaluates the similarity between 2 binary images (A and B) and is defined as follows:


$$DICE\left(A,B\right)=\frac{2\left|A\cap B\right|}{\left|A\right|+\left|B\right|}$$

Here, this index equals twice the number of elements common to both binary images (true positives) divided by the total number of elements in both images (2* True Positives + False Positives + False Negatives). This similarity quotient ranges between 0 and 1.

### Aortic Segmentation Pipeline: Image Preprocessing

Following data augmentation, all 825 sets of CT scans from 75 patients (284,624 CTA axial slices, 145,320 noncontrast CT axial slices) were preprocessed. Preprocessing steps included isotropic voxel conversion and down-sampling by a factor of 3.2 (512 × 512 × Z_initial/(i)_→160 × 160 × Z_initial/(f)_: Z_*f*_ *= Z*_*i*_ / 3.2). Here, *Z*_*i*_ and *Z*_*f*_ represents the number of axial slices within the study series before and after pre-processing. The down-sampled images were only used for aortic region of interest detection (ROI). The higher resolution images were used for aortic segmentation.

### Aortic Segmentation Pipeline: Aortic ROI Detection

Attention U-Nets A and D (*Attn U-Net A, D*, refer to Fig. 2A) were trained to segment the aorta from these decreased resolution, isotropic CTA and noncontrast CT images, respectively. These architectures were trained and evaluated using 26 patients (Table S1A, http://links.lww.com/SLA/C720). Aortic bounding boxes were generated from the model predictions. Two bounding boxes were generated from the contrast CT image [1. Thoracic (Thor.) and 2. Descending/ Abdominal Aorta (AAA)] and one was generated from the non-contrast CT image (1. Descending/ Abdominal Aorta (AAA)]. The ROIs derived from the bounding boxes served as the input data for aortic segmentation. Z_Thor_ or Z_AAA_ represent the number of axial slices within the thoracic aorta and descending aorta/AAA ROIs, respectively. All subsequent U-Nets (Attn *U-Nets B, C, and E*) were trained using the entire dataset of 75 patients (825 augmented augmented sets of images). This was done to expose the DL models to the diverse and complex aortic/aneurysmal morphology.

### Aortic Segmentation Pipeline: Aortic Segmentation

U-Nets B and C (*Attn U-Net B + C*, refer to Fig. 2A) were trained on the CTA ROIs to simultaneously segment the aortic lumen and WS regions of the thoracic and WS/ILT of abdominal aorta, respectively. U-Net E was trained on the non-contrast ROIs and was tasked to segment the abdominal aorta. For all 3 U-Nets, 3-fold cross-validation experiments were performed with a data-split of 50:25 patients between training and testing cohorts for each fold (Table S1B, http://link-s.lww.com/SLA/C720). Each fold consisted of 550 post-augmented images from 50 patients for training. The testing cohort consisted of 25 preaugmented images from 25 patients (testing cohort). There was no overlap between the train/validation and testing cohorts. Table S2, http://links.lww.com/SLA/C720 delineates all the U-Nets trained and evaluated in this study along with their learning parameters. Model training was performed simultaneously on a workstation with 2 × 11gb NVIDIA RTX 2080 TI graphics cards.

### Assessment of Model Accuracy Using Aortic Morphological Features

In addition to the DICE score, 1-,2- and 3-D measurements of aortic morphology were extracted from the aorta. These were used to evaluate the clinical validity of this high-resolution segmentation pipeline. We developed an in-house program in MATLAB to automate the extraction task. We assessed the inter-/intra- observer variation by comparing the algorithm output to the measurements manually extracted (ground truth) from the same CT images. From each patient, measurements were obtained both along the axial plane and the plane orthogonal to the aortic centerline. Six measurements were obtained from three slices [1. slice with the max anteroposterior (AP) diameter, 2–3.1 cm above and below the slice with the max AP diameter]. Max antero-posterior and transverse diameters were measured in each of the 3 slices. Coefficients of variation between the manual delineation and automatic methods are reported.

Maximum AP diameter (1-D) along the axial plane and axial area (2-D) of the aneurysmal region were automatically extracted from each 3-D image. Finally, 3-D measurements included spatial assessment of the lumen and ILT/WS from CTA images and of the total aortic volume from noncontrast CT images. All metrics were calculated on model predictions and ground truth (GT) segmentations using an in-house program in MATLAB. Bland Altman plots and correlation coefficient analysis assessed bias and the strength of association between the output of the DL models and the GT. Bias for all measurements was reported along with its 95% confidence interval (95% CI).

Second-order features including lumen and ILT/WS center-lines were calculated using an implementation of the homotopic thinning algorithm.^10^ Centerline deviation between model predictions and GT annotations was calculated using 1. Average Euclidean distances and 2. Hausdorff distance. The former calculates the distances between 2 closest points in the 2 centerlines. On the other hand, the Hausdorff metric reflects the upper bounds of the former.^11^ It is the greatest distance between a point in one centerline and the closest point in the adjacent line. Additionally, maximum diameter in planes perpendicular and orthogonal to the generated centerlines were extracted and compared between the ground truth measurements and the model predictions. Root-mean-square-error and percentage deviation were used to assess the similarity between the diameters orthogonal to the AAA centerline. This second-order feature assessment ensures the utility of model predictions for complex geometric/morphological analysis.

## Results

### CT Image Characteristics

Threefold cross-validation was used during training of the aortic segmentation pipeline. CT image characteristics between the training/validation and testing cohorts were extracted for each fold. Statistical comparison (2-tailed unpaired *t*-tests) between the training and testing cohorts for both CTA and noncontrast CT images across all 3 folds, revealed no significant differences. The CT image characteristics between the groups in fold 1 are summarized in Table S3, http://links.lww.com/SLA/C720.

### *Intra*- and *Inter*- Observer Variability Assessment

There were strong agreements for *inter- and intra-* observer measurements (GT) as measured by DICE (±SD) score and intra-class correlation coefficients from CTA and noncontrast CT images (Table S4, http://links.lww.com/SLA/C720). This supports the accuracy of the manual segmentations used for model training.

### Aortic Segmentation Pipeline: ROI Selection Accuracy

*Attn-U-Nets A and D* were trained and tested using the smaller cohort of 26 patients. These networks were tasked to extract the aortic volume from low-resolution isotropic CT images. Both model performances plateaued rapidly after 200 epochs of training. Figure S6, http://links.lww.com/SLA/C720 illustrates the evolving DICE score metric for the validation group during training of these architectures. The segmentation accuracies on the testing cohort for extracting the aortic mask from the CTA and noncontrast CT images were 93.4 ± 1.2% and 88.7 ± 4.2%, respectively. Implementing this network on the larger cohort allowed for accurate ROI selection of the aortic shape on all images.

### Aortic Segmentation Pipeline: Aortic Segmentation Accuracy

**Figure 3:**
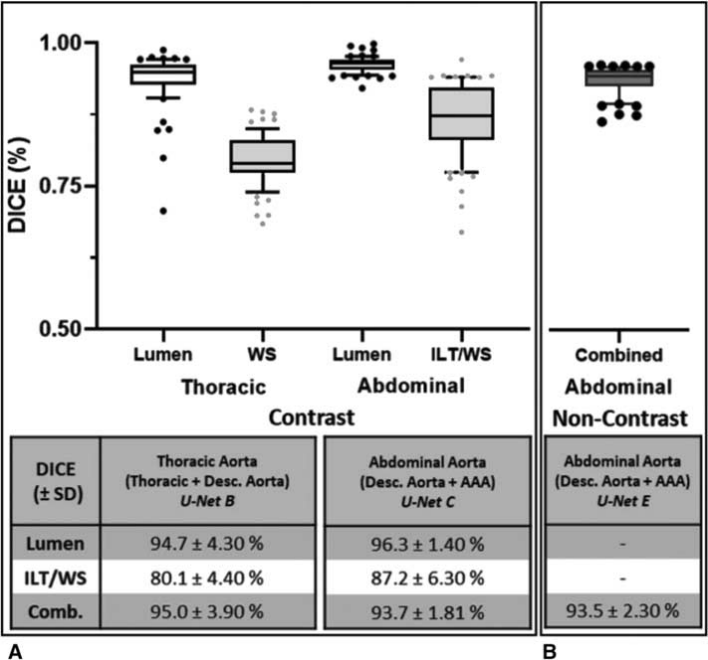
DICE accuracy of model predictions from CTA/noncontrast CT images compared against GT segmen-tations. A. DICE scores for the CTA-derived segmentations are divided into nonoverlapping ROIs (Thoracic Aorta [U-Net B] and Abdominal Aorta [U-Net C]). Scores for the lumen, thrombus/wall structure (ILT/WS) only and the combined aortic region are calculated. B. DICE scores for the non-contrast-derived segmentations of the combined aortic region are calculated for the descending aorta/AAA only [U-Net E].

Following ROI selection, threefold cross-validation was used to train the final segmentation models. Figure S7A, http://link-s.lww.com/SLA/C720 illustrates the evolving DICE score metric for the validation group during training of these architectures. Consequently, Figure 3 displays the performance of *Attn U-Nets B and C* on the ability to segment CTA images via the DICE score metric. The inner lumen DICE accuracy is comparable between the thoracic and the abdominal aorta regions. However, WS DICE accuracy is lower in the thoracic aorta compared to the abdominal aortic region. This is primarily because the thoracic aorta is mostly devoid of ILT and in most cases is a thin circular “ring” surrounding the lumen. Slight differences in this segmentation result in a relatively larger proportion of error, as compared to the abdominal region where WS differences will be proportionally less due to ILT presence.

For noncontrast CT images, threefold cross-validation was utilized to train *Attn U-Net E* (Fig. S7B, http://links.lww.com/SLA/ C720). Figure 3B displays the performance of *Attn U-Net E* to segment non-contrast CT images via the DICE metric. The aortic segmentation pipeline for a patient within the test cohort compared against GT annotations is illustrated in Figure S8, http://link-s.lww.com/SLA/C720.

### Aortic Segmentation Pipeline: First and Second Order Assessment of Aortic Morphology

Coefficients of variation (%CV) between manual and automatic measurements for the maximum AP and transverse diameters along the axial plane were 0.7 ± 0.05% and 1.1 ± 0.03%, respectively. Additionally, %CV between manual and automatic measurements for the maximum AP and transverse diameters along the plane orthogonal to the aortic centerline were 0.9 ± 0.04% and 1.4 ± 0.1%, respectively. This suggests high concordance between the manual and automatic methods in the calculation of clinical measurements and supports the use of the automatic extraction algorithm for subsequent steps.

**Figure 4:**
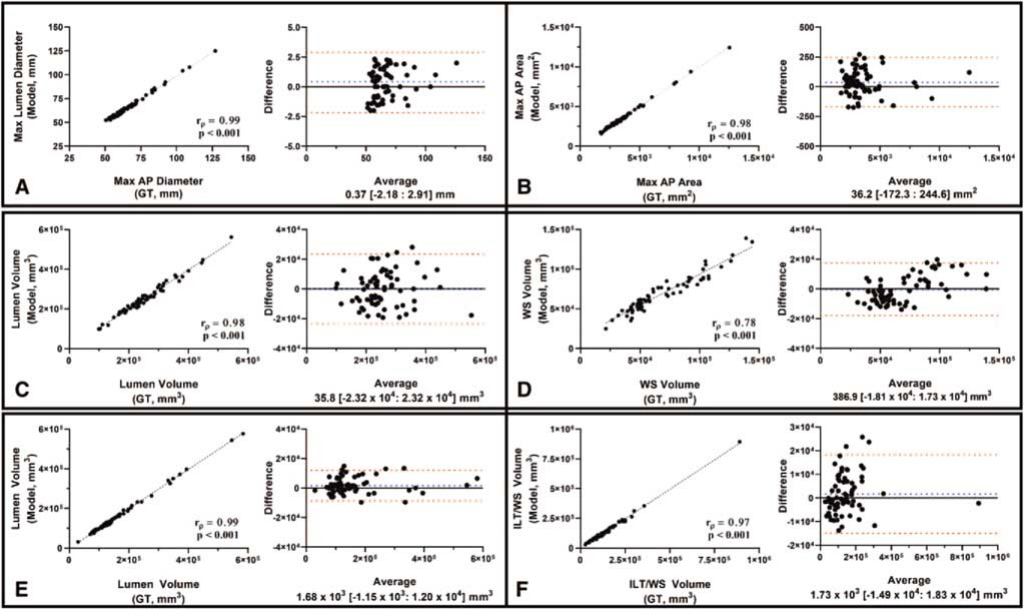
Bland-Altman plots and correlation-coefficient analysis comparing the 1-D (Max AP Diameter of AAA - A), 2-D (Max axial area of AAA - B), and 3-D (Lumen/WS volume of Thoracic Aorta – C-D, and lumen/WST/ILT volumes of the Abdominal Aorta - E-F) measurements derived from model predictions compared against those derived from the GT. This analysis was limited to volumes extracted from CTA images. Spearman correlation coefficients (rp) and P-values are indicated on the graphs.

Maximal AP diameter (*r*_*p*_ = 0.99, *P <* 0.001) and cross-sectional area (*r*_*p*_ = 0.98, *P <* 0.001) derived from model predictions of CTA images were very strongly correlated with manually derived measurements (Fig. 4A-B). The variability in diameter measurements was < 1.5 mm (1.2 ± 0.80%). Inner lumen (*r*_*P*_ = 0.98, *P <* 0.001) and WS (*r*_*P*_ = 0.78, *P* < 0.001) volumes of the thoracic aorta, derived from the output of U-Net B, were strongly correlated with those obtained from the GT annotations (Fig. 4C-D). The variability in the WS volume measurements (12.40% ± 8.10%) were noticeably greater than for the lumen (3.90% ± 2.64%) in the thoracic aorta. This is inherently linked with its thin circumferential distribution in the thoracic aorta.

**Figure 5:**
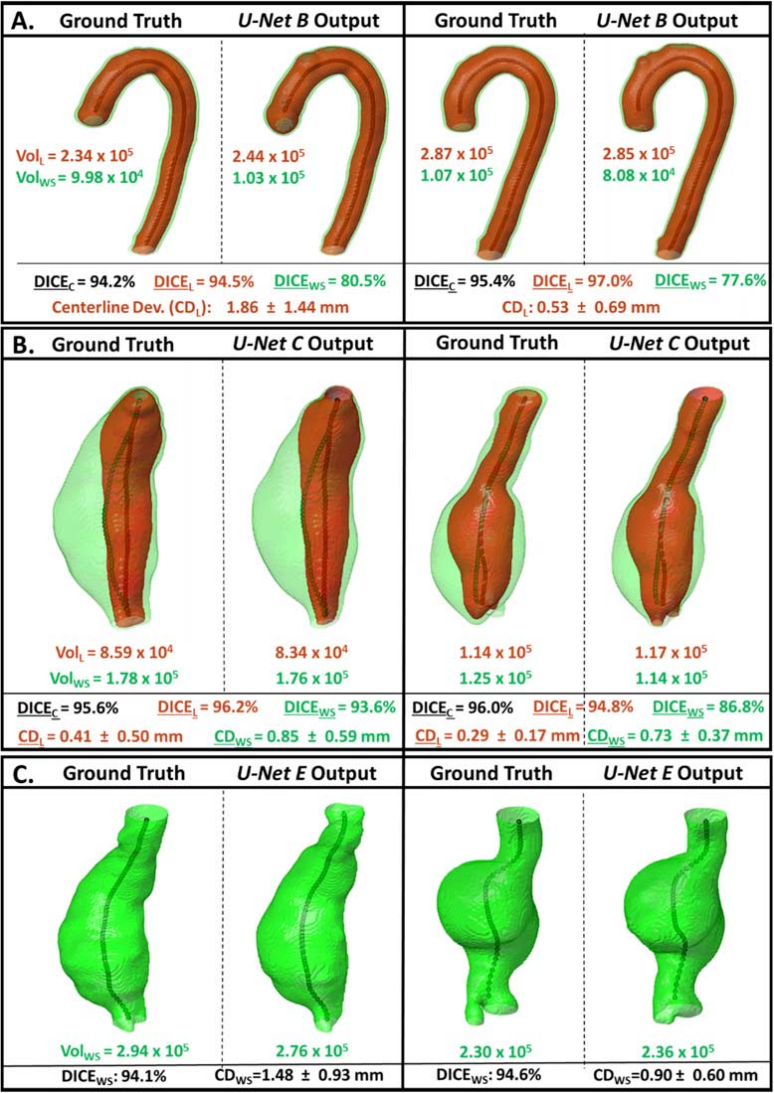
Model predictions of the thoracic (A) and abdominal (B) aortic regions from CTA images and the abdominal region from noncontrast CTimages (C) within the testing cohort are displayed alongside labelled GT masks. WS and Lumen volumes, when available, are indicated next to each segmentation. DICE scores for the lumen (black), WS (grey), and the combined aortic predictions are indicated for each patient. The difference in centerlines derived from the lumen (CDL +/– SD) and wall structure masks (CDWS +/– SD) are indicated as average Euclidean distance deviation. CDL indicates Centerline Deviation of Lumen;SD, Standard Deviation.

Inner lumen (*r*_*p*_ *=* 0.99, *P* < 0.001) and ILT/WS (*r*_*p*_ *=* 0.97, *P* < 0.001) volumes from the abdominal aorta, derived from the output of U-Net C, were very strongly correlated with manually-extracted volumes (Fig. 4E-F). In this case, the ILT/WS variability in the abdominal region (5.50 ± 3.01%) is lower than that in the thoracic aorta (Table 1A). Model predictions of the thoracic (*U-Net B*) and the abdominal aorta (*U-Net C*) from 4 patients in the testing cohort alongside their GT masks are shown in Figure 5A-B.

**Table 1:** Clinical Assessment of Segmented Volumes From CTA and Noncontrast Images

CTA	% Difference (±SD)	% Difference (±SD)	Noncontrast CT	% Difference (±SD)
Thoracic aorta *(U-Net B)*		Abdominal Aorta / AAA *(U-Net C)*	Abdominal Aorta / AAA *(U-Net E)*	
Max AP diameter	**—**	1.20 ± 0.80%	Max AP Diameter	1.67 ± 1.10%
Max axial area	**—**	2.96 ± 2.45%	Max Axial Area	3.60 ± 3.02%
Lumen volume	3.90 ± 2.64%	2.90 ± 2.60%	AAA Volume	1.67 ± 1.10%
WS volume	12.40 ± 8.10%	5.50 ± 3.01%		
	Deviation (Euclidean Distance, ± SD)	Deviation (Euclidean distance, ±SD)		Deviation (Euclidean Distance, ±SD)
				
Lumen				
Centerline Dev.	1.07 ± 0.67 mm	0.85 ± 0.52 mm	AAA	
Hausdorff dist.	2.60 ± 2.02 mm	2.54 ± 1.98 mm	Centerline deviation	1.94 ± 1.00 mm
WS				
Centerline Dev.	1.64 ± 0.80 mm	1.04 ± 0.57 mm		
Hausdorff dist.	2.84 ± 1.59 mm	2.73 ± 1.73 mm	Hausdorff distance	3.58 ± 2.08 mm

Furthermore, the similarity in the lumen and ILT/WS center-lines generated from the model predictions and GT annotations is highlighted in Table 1A. Centerline deviations within the thoracic aorta are greater than those observed within the abdominal aorta. This may be due to the difficulty in delineating the border between the aorta and branching arteries within the thoracic region. Modelderived segmentations of these outlets may affect centerline properties greater than other metrics. However, the average Euclidean distance deviation is less than 2 mm for the thoracic aorta in 89% of cases (67/75) and for the abdominal aorta in 92% of cases (69/75).

**Figure 6:**
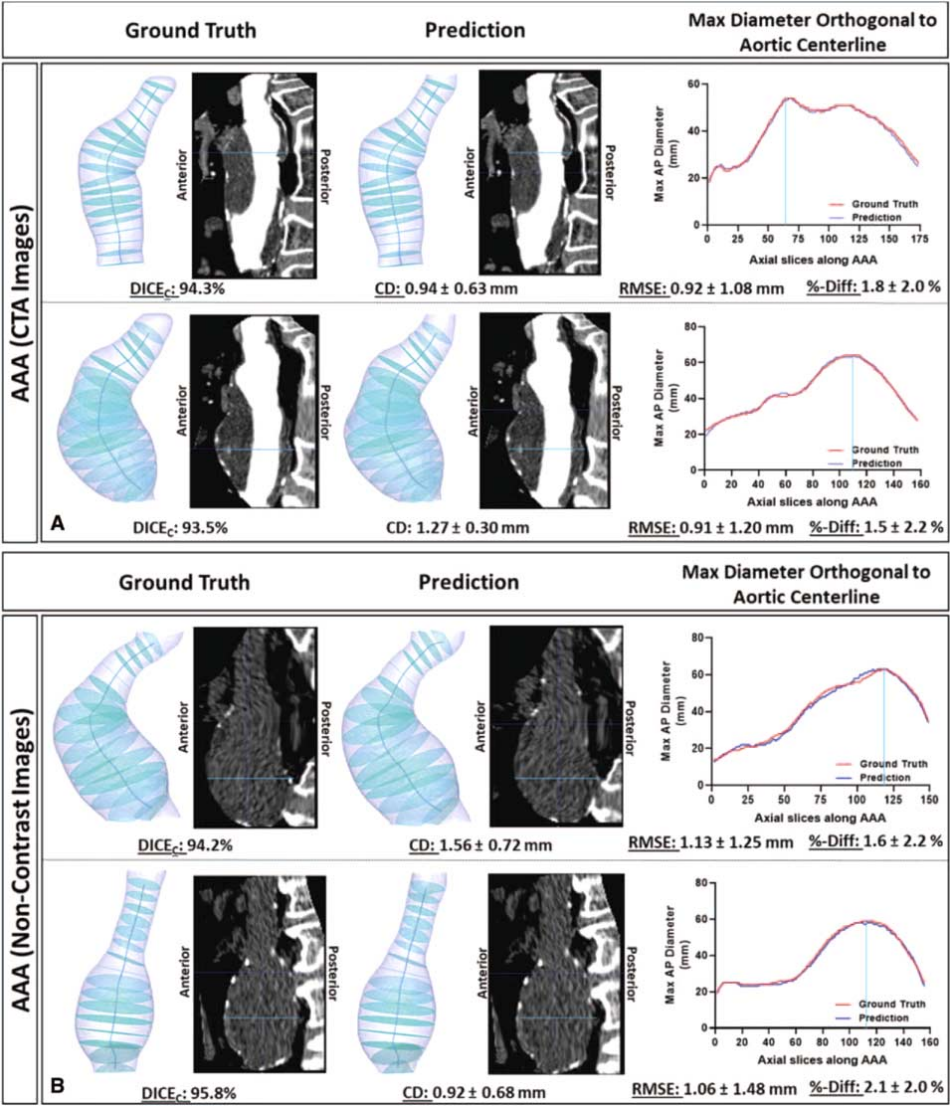
Maximum AAA Diameter profiles along planes orthogonal to AAA centerline. Profiles were generated from ground truth and model predictions of AAA volumes derived from contrast-enhanced (Panel A) and noncontrast (Panel B) CT images. The CT images displayed are the straightened views through the sagittal plane (realigned using the centerline). Corresponding DICE scores, average Euclidean distance deviations between centerlines, RMSE and %-difference of diameter profiles are indicated.

The generated centerlines allow for the automatic calculation of max AP diameter along planes orthogonal to the aortic centerline. The resulting diameter profiles between ground truth and predictions were compared and showed a RMSE of 1.45 ± 1.65 mm, which is equivalent to a percentage difference of 2.3 ± 1.1%. Figure 6A illustrates 2 examples of AAAs with planes orthogonal to centerline. These planes were used to generate the re-aligned or straightened view of the AAA. Corresponding DICE scores and average Euclidean distance deviations between centerlines are indicated. Maximum diameter profiles are illustrated for both ground truth and model predictions. These results support the clinical strength of this automatic segmentation platform for CTA images.

**Figure 7:**
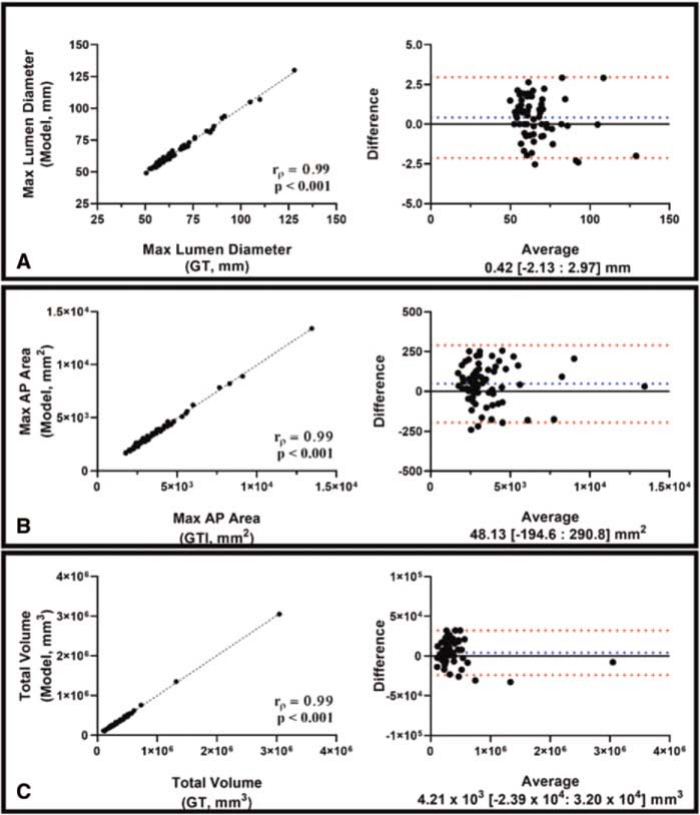
Bl and-Altman plots and correlation-coefficient analysis comparing the 1-D (Max AP Diameter of AAA - A), 2-D (Max axial area of AAA - B), and 3-D (Total volume of AAA - C) measurements from model predictions compared against those derived from GT. This analysis was limited to volumes extracted from noncontrast CT images. Spearman correlation coefficients (*r*_p_) and *P*-values are indicated on the graphs.

Maximal AP diameter (*r*_*p*_ = 0.99, *P <* 0.001), cross-sectional area (*r*_*p*_ = 0.99, *P <* 0.001) and volume (*r*_*p*_ = 0.99, *P <* 0.001) measurements extracted from the model predictions of non-contrast CT images are very strongly correlated with those derived from the GT segmentations (Fig. 7). The variability in extracting these measurements from noncontrast CT-derived segmentations is like that of CTA-derived annotations (Table 1B). Model predictions from 2 patients within the testing cohort are illustrated in Figure 5C. The resulting diameter profiles between ground truth and predictions were compared and showed a RMSE of 2.11 ± 1.32 mm, which is equivalent to a percentage difference of 2.8 ± 2.1%. Figure 6B illustrates 2 examples of AAAs with planes orthogonal to centerline. This study shows for the first time the ability to segment the aneurysmal aorta from noncontrast CT images at a level comparable to a human observer.

## Discussion

This study describes a fully automatic and high-resolution algorithm able to extract the aortic volume from both CTA and noncontrast CT images at a level superior to that of other currently published methods.^12,13^ High accuracy of our segmentation pipeline was supported by the DICE score metric between model predictions and ground truth annotations for both the thoracic and abdominal aorta. However, this metric, which evaluates the similarity between 2 binary images by evaluating the extent of pixel overlap, has its limitations. In cases of small volumes, minimal changes lead to lower DICE score percentages. This is especially true in the outer wall structure region of the thoracic aorta. This region is usually a thin, circumferential ring around the aortic inner lumen. Small variations within this small volume lead to relatively greater variability and lower DICE scores. Concurrently, if there are small but critical errors in a relatively large volume, the DICE score would remain elevated, but the clinical utility of the image would be diminished. However, this is a common way to compare the performance of segmentation algorithms across methods.

To address the limitations of DICE score metric, clinical utility was demonstrated by comparing first and second-order measurements, which are important parameters for characterizing AAA progression. Extracting max AP diameter measurements enables the calculation of growth during surveillance and determines timing of surgery.^14,15^ Assessing the accuracy of diameter extraction is essential to integrate this DL platform with current methods of AAA management. Cross-sectional area (2-D) has been shown to have the lowest variability in assessing aneurysm change and supplements the 1-D diameter measurements.^16^ Evolution of 3-D, especially thrombus volume, and second order indices, including centerline is linked to AAA progression, rupture risk and the incidence of adverse cardiovascular events.^17,18^ This automatic method of volume extraction can be used to standardize current methods of aneurysmal disease management and sets the foundation for subsequent complex geometric analysis. Furthermore, the proposed pipeline can be extended to other vascular pathologies.

Before the advent of machine-learning approaches, AAA segmentations were performed using intensity-based semi-automatic lgorithms (eg, level-sets, active shape models and graph cut methods).^12,19–21^ Their primary drawback was the failure to accurately detect the aneurysm’s outer boundary as its intensity is like that of adjacent structures. Although these models may provide good results, there are significant limitations that prevent clinical implementation. These methods are semi-automatic and require significant model optimization. Furthermore, these methods require complex user-input (ex. prior lumen segmentations/centerlines), and are highly data-set dependent.^12,19^ The latter limits model robustness and generalizability.

Recently, DL methods on CTAs have been proposed to tackle this problem without encountering many of the limitations of their predecessors. Variations on Deep Belief and U-net based networks have been used to segment the infra-renal region of the aorta.^13,22^ Unfortunately, many of these networks are limited to 2-D inputs (axial CT slices), which may fail to appropriately capture the aneurysm’s 3D geometry. The accuracy and reproducibility of these models is like that of earlier methods as they are trained and validated on small data sets. Lopez-Linares et al recently proposed a Holistically-Nested Edge Detection network trained in both 2D and 3D that out-performs currently available methods in both pre and post operative AAA segmentation.^23^ However, this method is limited to single-class segmentation of the aneurysmal wall and performs poorly with small aneurysms and those with a small thrombus burden.

Current convolutional neural network architectures can capture semantic contextual information by generating a coarse feature-map grid through iterative down-sampling of the input. Features on this coarse map represent location and relationship between structures/tissues at the organ level. However, these architectures struggle to capture small target objects with increased shape variability. This is especially important for pathological vascular cases. Integrating attention gates, which is commonly used in natural image analysis and classification tasks, into this architecture has shown promise in focusing on target structures without the need for additional train-ing.^6^ These attention gates can suppress predictions in irrelevant background region and can be trained simultaneously with the underlying network using standard back-propagation methods. The strength of this attention-based U-Net has been documented on the segmentation of abdominal structures^6^; however, its role in aortic segmentation has never before been evaluated. Its superior segmentation performance for aneurysmal segmentation rationalized its incorporation within the full aortic segmentation pipeline.

This is the first time a DL method is used to isolate the aorta/AAA from a noncontrast CT scan. This allows for the extraction of complex morphological information from noncontrast images. Furthermore, the same methodology underpinning this work can be extended to enable automatic segmentation of other structures with or without the use of IV contrast agents.

Although CTAs provide unique insight into aneurysm morphology and the vascular tree, it is not without its disadvantages. Administration of contrast requires needle insertion, which is associated with multiple complications including inadvertent arterial puncture and contrast leak from veins causing skin irritation/damage. Additionally, contrast agents are nephrotoxic and have a 10% incidence of acute kidney injury (contrast-induced nephropathy) after use. This is a problem within the elderly population, who either have decreasing baseline renal function or concomitant chronic kidney disease. Given that a large sub-cohort of patients with aortic aneurysmal disease may have diagnosed renal disease, this study highlights the necessity to re-evaluate the role of noncontrast CT imaging for the management of aneurysmal disease.

## Conclusions

In this study, a novel automated pipeline was developed to enable high resolution segmentation of blood vessels using deep learning approaches. This clinically validated pipeline enables automatic extraction of morphologic features of blood vessels and can be applied for research and potentially for clinical use.

## Supplementary Material

SUPPLEMENTARY MATERIAL
