# Supplementary material for: A Deep Learning Pipeline to Automate High-Resolution Arterial Segmentation With or Without Intravenous Contrast
Source: Ann Surg. 2020 Nov 23;276(6):e1017–27. doi: 10.1097/SLA.0000000000004595 (PMC9645535; doi:10.1097/SLA.0000000000004595)
Supplement: SUPPLEMENTARY MATERIAL [file sla-276-e1017-s001.pdf]

Supplement: U-Net Model Selection and Evaluation

Model Selection

We hypothesized that the 3D U-Net with attention gating would outperform the generic 3D U-Net(1) for segmentation of the aorta. A smaller cohort of twenty-six patients was used to evaluate the performance of these networks for aneurysmal segmentation. As seen in ***Table S1a***, post-augmented scans were split into training (n_{train} = 10 patients, 110 augmented scans), and validation (n_{valid} = 3 patients, 33 augmented scans) groups. This was done to avoid data leakage between the training and validation groups. Training parameters utilized in this experiment are highlighted in ***Table S2***. The validation group was used at the end of each training epoch to gauge model performance during training and fine-tune model hyperparameters. The remaining 13 patients formed the testing cohort (n_{test} = 13). Model predictions of the testing cohort images were evaluated using the DICE metric to determine the impact of attention-gating for aneurysmal segmentation. Additional morphological metrics were calculated. A student t-test was used to compare the performance of the two networks. The model exhibiting superior performance was then implemented in the automated aortic segmentation pipeline.

Model Evaluation for AAA segmentation: Attention-based 3D-U-Net vs 3D-U-Net

To assess the benefit of attention-gating for AAA segmentation, the performance of an attention-based 3D U-Net was compared against that of a generic 3D U-Net. For this evaluation, a subset of 26 cases were used from the larger cohort. These images were split as per Table 1a into train, validation and test cohorts. **Fig. S4** illustrates the evolving DICE score metric for the validation group during model training. During the training of the Attention-based U-Net, the overall DICE score plateaus at 95.3% after 1000 epochs (Inner Lumen: 97.4%, Wall structure: 89.2%). On the other hand, the performance of the control 3D U-Net plateaus at approximately 91.8% (Inner Lumen: 96.4%, Wall structure: 87.2%).

Segmentation of the testing cohort was used to evaluate model performance. Model output was compared against the manually segmented GT images utilizing the DICE score metric. The results of this analysis are found in ***Table S5a***. The accuracy of the Attention-based U-Net in extracting the ILT/wall structure of the aneurysm is significantly superior to that of the generic 3D- U Net. Additionally, the results show that the aneurysm output produced by attention-based U-Net has a stronger correlation to the GT segmentations for all metrics evaluated than that of the generic 3D U-Net (***Table S5b***). Similarly, the Bland-Altman plots for the attention-based U-Net indicate smaller biases with notably smaller bounds (95% confidence interval) when compared against the 3D U-Net outputs (**Fig S4)**.

Comparing to the manual segmentation (ground truth), the difference of AP diameter as measured by the *Attn-U-Net* and *3D-U-Net* is 0.66 ± 0.56 mm and 2.30 ± 1.36 mm, respectively ($p < 0.01$). The attention-based 3D U-Net architecture was able to measure the maximum AP diameter to within < 1mm accuracy in 77% (or 10/13) of the cases, as compared to the standard 3D U-NET which can achieve this accuracy margin in 15% (or 2/13) of cases. Similar levels of accuracy were documented when evaluating the inner lumen and ILT/WS volumes. This rationalizes the incorporation of the attention-gating unit into the segmentation pipeline. Example model outputs within the test set are shown in **Fig. S5** along with their respective gold standards and DICE similarity scores.

Supplement: Tables

Table S1: Patient allocation between the training, validation and testing cohorts.

**A**	**Model Selection and Aortic ROI Detection**		
	Training Cohort (n_{train})	Validation Cohort (n_{valid})	Testing Cohort (n_{test})
**Patients**	10	3	13
**Post-Augmented Scans**	110	33	-
**B**	**Aortic Segmentation**		
	n_{train}	n_{valid}	n_{test}
**Patients**	45	5	25
**Post-Augmented Scans**	495	55	-

Table S2: U-Nets trained for model selection and the segmentation pipeline with learning parameters.

**Model**	**Epochs**	**Learning Rate**	**Weight Decay**	**Batch Size**	**Task**	**Implementation**
Model Selection						
U-Net	1000	$1.0 * 10^{-3}$	$1.0 * 10^{-6}$	2	Multi-Class AAA Segmentation	*Attn U-Net* vs *U-Net* for AAA Segmentation
Attn U-Net	1000	$1.0 * 10^{-3}$	$1.0 * 10^{-6}$	2		
Aortic Segmentation Pipeline						
Attn U-Net A	600	$1.0 * 10^{-3}$	$1.0 * 10^{-6}$	2	Aortic Segmentation from low-resolution isotropic CTA	Aortic ROI Detection (Contrast)
Attn U-Net B	750	$1.0 * 10^{-3}$	$1.0 * 10^{-6}$	2	Multi-Class Aortic Arch Segmentation from high-resolution isotropic CTA	Aortic Segmentation (Contrast)
Attn U-Net C	1000	$1.0 * 10^{-3}$	$1.0 * 10^{-6}$	2	Multi-Class Descending Aorta + AAA Segmentation from high-res. isotropic CTA	Aortic Segmentation (Contrast)
Attn U-Net D	600	$1.0 * 10^{-3}$	$1.0 * 10^{-6}$	2	Aortic Segmentation from low-resolution isotropic Non-Contrast CT	Aortic ROI Detection (Non-Contrast)
Attn U-Net E	1000	$1.0 * 10^{-3}$	$1.0 * 10^{-6}$	2	Aortic Segmentation from high-resolution isotropic Non-Contrast CT	Aortic Segmentation (Non-Contrast)

Table S3: Image characteristics within the training and external test cohorts (Fold 1).

		Training Cohort (n = 50)		Test Cohort (n = 25)		p-value
Contrast	25th Percentile HU [95% CI]	-1008	[-1003 -1014]	-1006	[-1002 -1010]	0.42
	Mean HU [95% CI]	-587	[-646.1 -527.6]	-568.4	[-610.2 -526.6]	0.48
	75th Percentile [95% CI]	-67	[-45.8 -85.8]	-54.1	[-40.8 -67.4]	0.18
	Standard Deviation [95% CI]	484.1	[475.0 493.2]	490.6	[485.1 495.9]	0.08
	Voxel Length [95% CI]	0.81 mm	[0.76 0.86]	0.83 mm	[0.79 0.87]	0.50
	Voxel Height [95% CI]	0.81 mm	[0.76 0.86]	0.83 mm	[0.79 0.87]	0.50
	Voxel Thickness	1.25 mm		1.25 mm		-
	KiloVoltage Peak (kVP)	120		120		-
	Exposure Time [95% CI]	434	[359.2 508.8]	474.2	[369.3 579.0]	0.50
	X-Ray Tube Current (mA)	265.3	[105.3 425.7]	299.1	[140.2 467.4]	0.32
Non-Contrast	25th Percentile HU [95% CI]	-1009	[-1005 -1013]	-1005	[-1002 -1008]	0.07
	Mean HU [95% CI]	-565	[-524.3 -606.7]	-550.6	[-589.4 -511.9]	0.55
	75th Percentile [95% CI]	-53.4	[-39.9 -66.9]	-46.8	[-62.2 -31.3]	0.49
	Standard Deviation [95% CI]	483.2	[476.0 490.0]	484.8	[479.8 489.9]	0.69
	Voxel Length [95% CI]	0.80 mm	[0.75 0.85]	0.82 mm	[0.78 0.86]	0.58
	Voxel Height [95% CI]	0.80 mm	[0.75 0.85]	0.82 mm	[0.78 0.86]	0.58
	Voxel Thickness	2.5 mm		2.5 mm		-
	KVP	120		120		-
	Exposure Time [95% CI]	457.3	[435.2 467.8]	462.2	[448.3 475.0]	0.63
	X-Ray Tube Current (mA)	355.6	[221.8 489.4]	367.5	[209.7 525.3]	0.49

Table S4: DICE score and intra-class correlation for *Intra-*/*Inter-* operator segmentations (*p < 0.001).

Region		*Intra-*		*Inter-*	
		DICE ± SD (%)	ICC	DICE ± SD (%)	ICC
Contrast DICE ± SD (%)	Inner Lumen	98.0 ± 0.2 %	1.000 *	96.5 ± 0.4 %	0.995 *
	Entire Aorta	97.8 ± 0.5 %	0.989 *	96.1 ± 0.6 %	0.981 *
	Wall Structure + ILT Only	95.1 ± 0.8 %	0.981 *	93.1 ± 0.9 %	0.974 *
Non-Contrast	Entire Aorta	96.8 ± 0.4%	0.988 *	95.2 ± 0.8 %	0.977 *

Table S5: Aortic Segmentation Accuracy of the Attn U-Net vs 3D-U-Net.

	**Attn U-Net vs. Ground Truth**	**3D U-Net vs. Ground Truth**	
**Region**	**DICE (± SD)**	**DICE (± SD)**	**p-value**
**Inner Lumen**	96.8 ± 1.2 %	94.4 ± 1.4 %	0.76
**Entire AAA**	94.8 ± 0.9 %	89.5 ± 1.1 %	**0.01**
**Wall structure + ILT Only**	88.2 ± 1.9 %	85.2 ± 1.9 %	**< 0.01**
	**% Difference (± SD)**	**% Difference (± SD)**	
**Max AP Diameter**	1.1 ± 0.9 %	3.8 ± 2.2 %	**< 0.01**
**Max Axial Area**	2.5 ± 1.6 %	5.0 ± 2.8 %	**0.01**
**IL Volume**	0.3 ± 0.9 %	1.4 ± 1.8 %	**0.02**
**ILT/WS Volume**	3.1 ± 2.1%	8.8 ± 8.4 %	**0.01**

Supplement: Figures

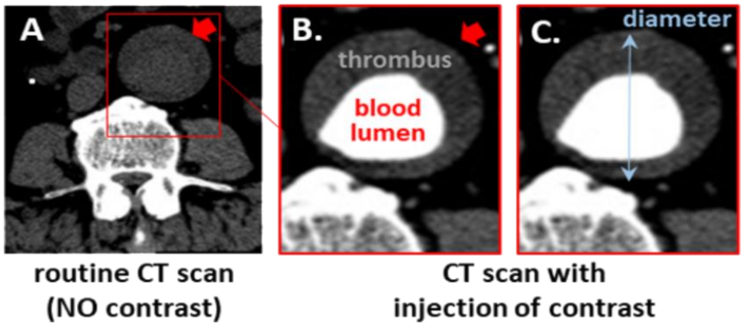

Fig S1: Axial slice through an abdominal aortic aneurysm from a non-contrast CT and CTA image.

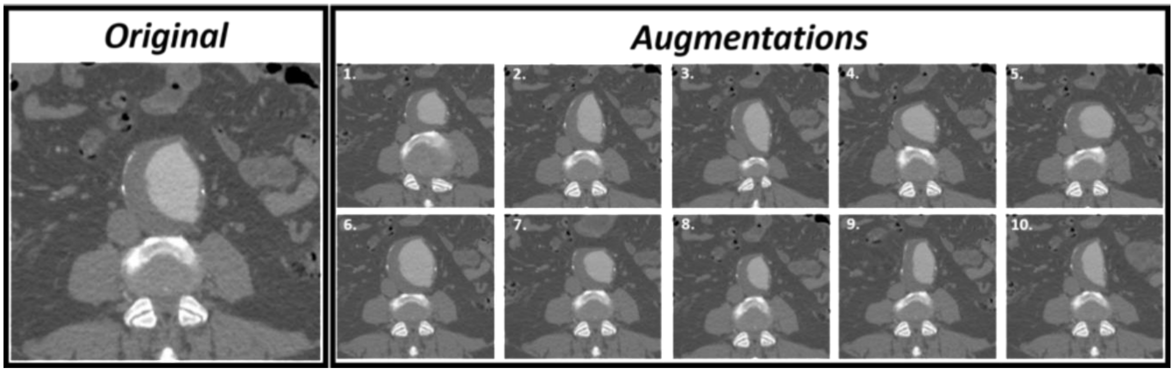

Fig S2: Axial slice (***Original***) is augmented 10:1 using divergence transformations.

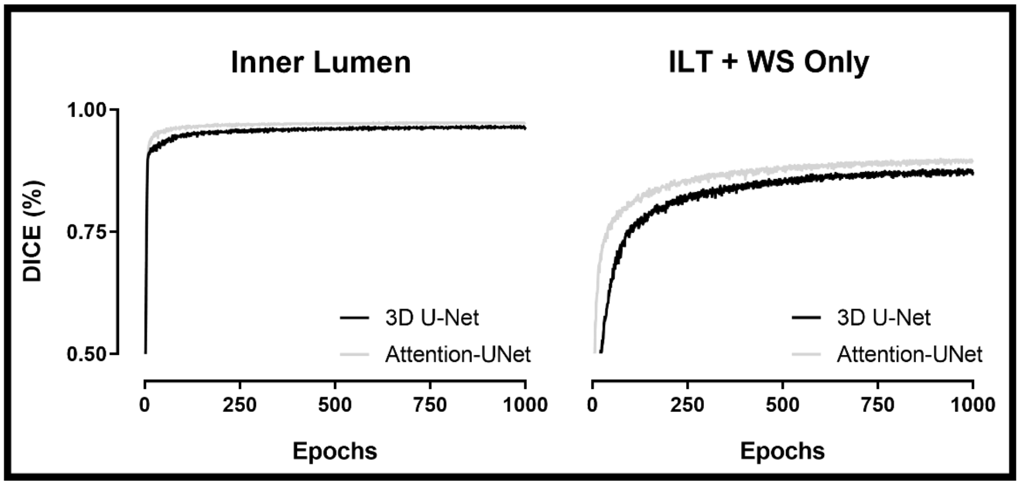

Fig S3: *Attention U-Net vs 3D U-Net for AAA Segmentation*. Training Paradigm for the Validation cohort. The Attention U-Net and generic 3D U-Net were trained for a total of 1000 epochs. Model outputs were assessed at each iteration and were compared against the GT segmentation using the DICE metric.

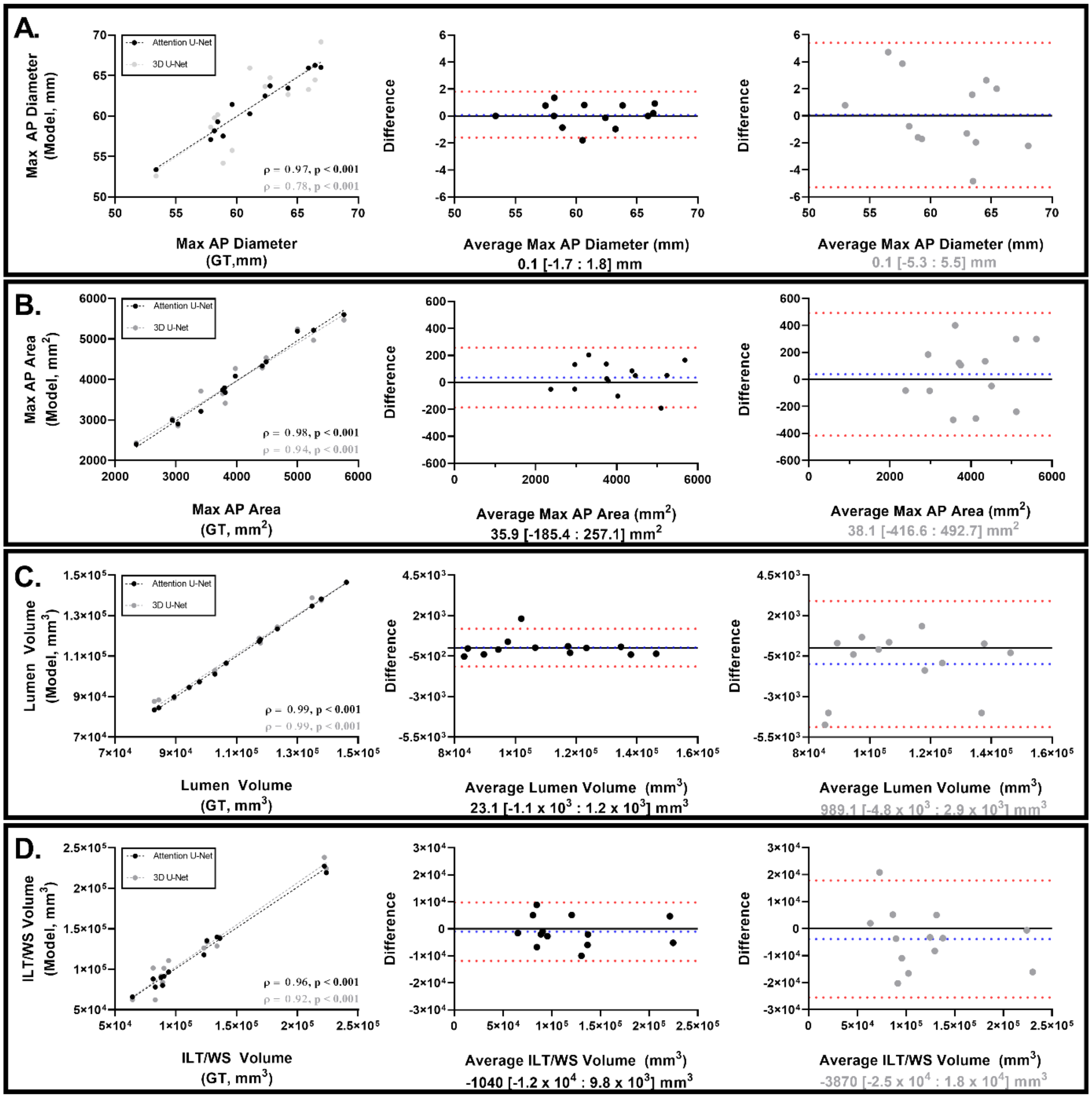

Fig S4: Intra-class correlation and Bland-Altman plot analysis comparing 1-,2-and 3-D measurements obtained from the model predictions and the GT segmentations. Bias [95% CI] within the respective models (Attention U-Net, 3D U-Net) is displayed for each measurement.

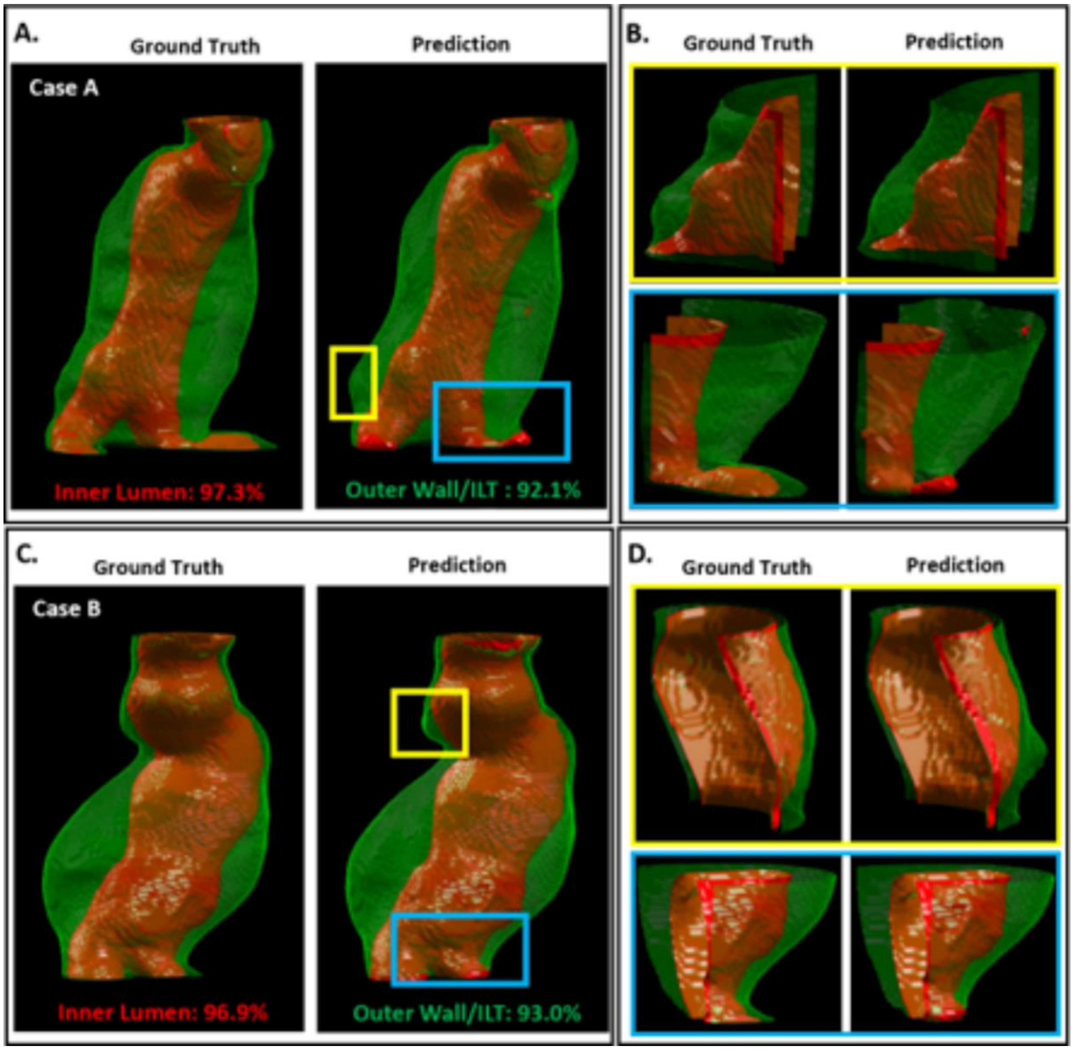

Fig S5: Attention-based 3D- U-Net outputs from two patients in the testing cohort **(A,C)** with the labelled GT masks. DICE scores for both the inner lumen and wall structure predictions are indicated for each patient. Various points of discrepancy within the two predictions are highlighted **(B,D)**.

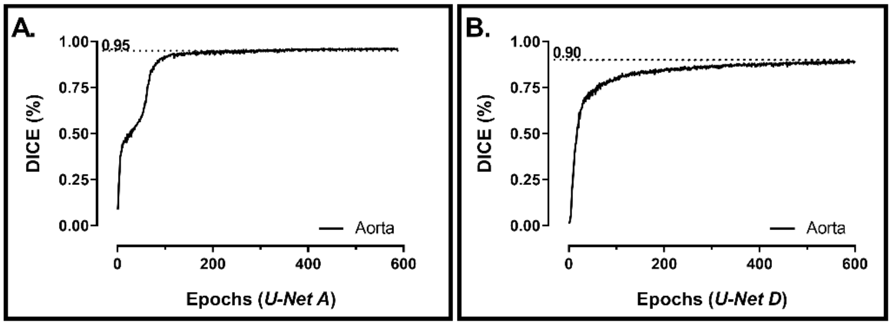

Fig S6: Training paradigm of the Attention-based U-Nets for Aortic ROI detection from CTA images (**A**) and Non-Contrast (**B**) CT Images **A.** *Attn U-Net A* was trained for 600 epochs on down-sampled isotropic CT images. A. *Attn U-Net D* was trained for 600 epochs on down-sampled isotropic non-contrast images.

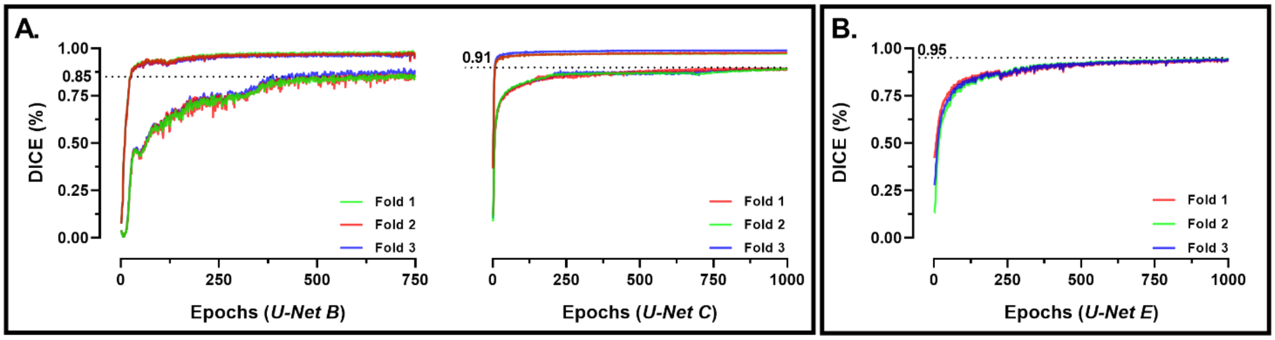

Fig S7: Training paradigm of the Attention-based U-Nets for Aortic Segmentation from CTA and Non-Contrast images. **A.** U-Nets B and C were trained on the contrast-enhanced ROIs derived from U-Net A – thoracic Aorta and descending aorta/AAA ROIs. **B.** U-Net E was trained for 1000 epochs on the non-contrast ROIs derived from U-Net D. A 3-fold cross-validation approach was used this training step.

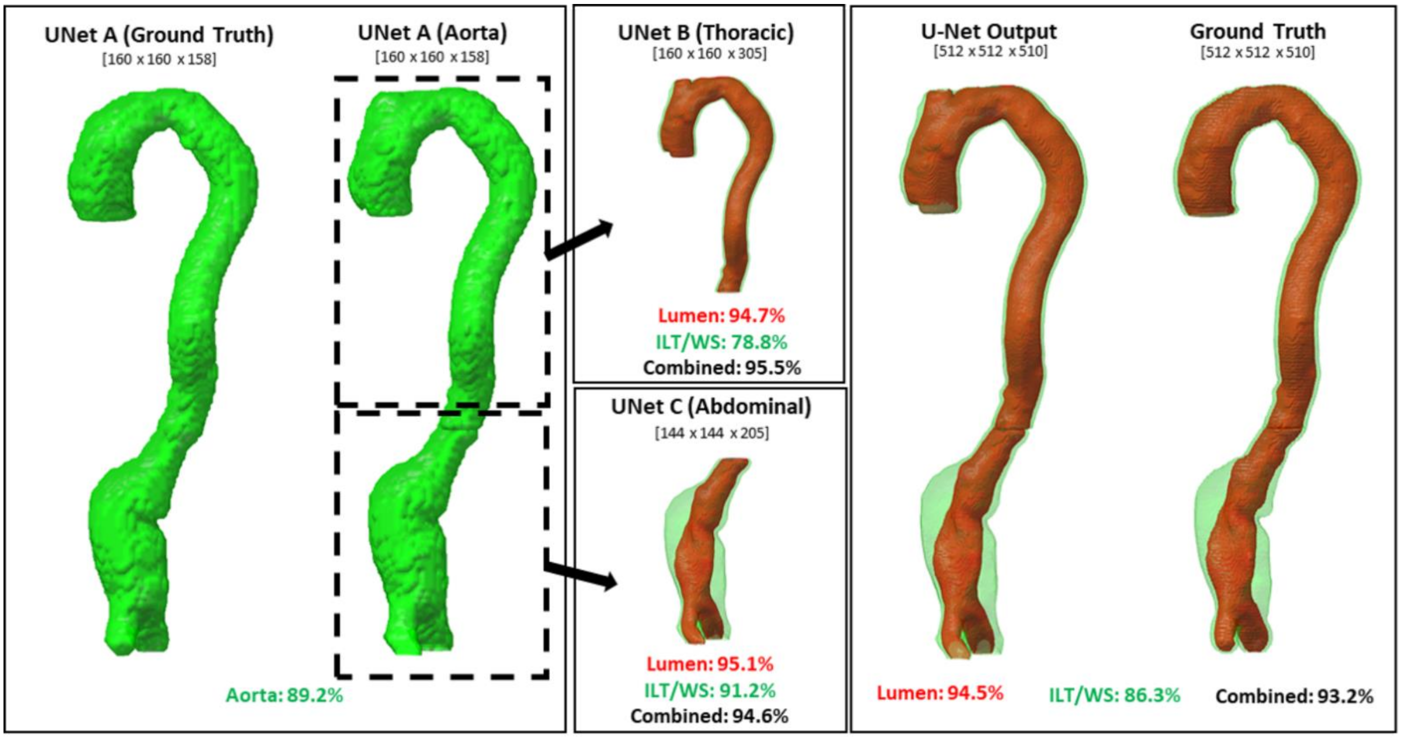

Fig S8: Aortic segmentation pipeline (*Attn U-Nets A – C)* for a patient within the testing cohort. **A.** *Attn U-Net A* identified the aortic structure from down-sampled images and was the basis for thoracic and abdominal/AAA aortic ROI detection. **B-C.** *Attn U-Nets B + C* identified the lumen and WS/ILT predictions for their indicated region. **D.** Region predictions were combined to assess overall accuracy.

References

1. Çiçek Ö, Abdulkadir A, Lienkamp SS, Brox T, Ronneberger O. 3D U-Net: Learning Dense Volumetric Segmentation from Sparse Annotation. arXiv e-prints, 2016:arXiv:1606.06650.
